# Enhancing European capabilities for application of multi-omics studies in biology and biomedicine space research

**DOI:** 10.1016/j.isci.2023.107289

**Published:** 2023-07-12

**Authors:** Aránzazu Manzano, Silvio Weging, Daniela Bezdan, Joseph Borg, Thomas Cahill, Eugénie Carnero-Diaz, Henry Cope, Colleen S. Deane, Timothy Etheridge, Stefania Giacomello, Gary Hardiman, Natalie Leys, Pedro Madrigal, Felice Mastroleo, F. Javier Medina, Jakub Mieczkowski, Manuel A. Fernandez-Rojo, Keith Siew, Nathaniel J. Szewczyk, Stephen B. Walsh, Willian A. da Silveira, Raúl Herranz

**Affiliations:** 1 Centro de Investigaciones Biológicas Margarita Salas (CSIC), 28040 Madrid, Spain; 2 University of Halle, 06108 Halle (Saale), Germany; 3 Institute of Medical Genetics and Applied Genomics, University of Tübingen, 72076 Tübingen, Germany; 4 NGS Competence Center Tübingen (NCCT), University of Tübingen, Tübingen, Germany; 5 Yuri GmbH, Meckenbeuren, Germany; 6 Department of Applied Biomedical Science, Faculty of Health Sciences, University of Malta, 2080 Msida MSD, Malta; 7 Faculty of Medicine, Health and Life Sciences, School of Biological Sciences, Institute for Global Food Security, Queen’s University Belfast, Belfast BT7 1NN, UK; 8 Institut Systématique, Evolution, Biodiversité (ISYEB), Muséum National d’Histoire Naturelle, Sorbonne Université, CNRS, EPHE, UA, 75005 Paris, France; 9 School of Medicine, University of Nottingham, Derby DE22 3DT, UK; 10 Human Development & Health, Faculty of Medicine, University of Southampton, Southampton General Hospital, Southampton, UK; 11 Department of Sport and Health Science, College of Life and Environmental Sciences, University of Exeter, Exeter EX1 2LU, UK; 12 Science for Life Laboratory, KTH Royal Institute of Technology, 17165 Stockholm, Sweden; 13 Microbiology Unit, Belgian Nuclear Research Centre, SCK CEN, 2400 Mol, Belgium; 14 European Molecular Biology Laboratory, European Bioinformatics Institute, EMBL-EBI, Hinxton CB10 1SD, UK; 15 3P-Medicine Laboratory, Medical University of Gdansk, Gdansk, Poland; 16 Hepatic Regenerative Medicine Lab, Madrid Institute for Advanced Studies in Food, 28049 Madrid, Spain; 17 University College London, London NW3 2PF, UK; 18 Ohio Musculoskeletal and Neurological Institute, Heritage College of Osteopathic Medicine, Ohio University, Athens, OH 45701, USA; 19 Department of Biological Sciences, School of Health, Science and Wellbeing, Staffordshire University, Stoke-on-Trent ST4 2DF, UK; 20 International Space University, 67400 Illkirch-Graffenstaden, France

**Keywords:** Space medicine, Genomics, Proteomics, Space sciences

## Abstract

Following on from the NASA twins’ study, there has been a tremendous interest in the use of omics techniques in spaceflight. Individual space agencies, NASA’s GeneLab, JAXA's ibSLS, and the ESA-funded Space Omics Topical Team and the International Standards for Space Omics Processing (ISSOP) groups have established several initiatives to support this growth. Here, we present recommendations from the Space Omics Topical Team to promote standard application of space omics in Europe. We focus on four main themes: i) continued participation in and coordination with international omics endeavors, ii) strengthening of the European space omics infrastructure including workforce and facilities, iii) capitalizing on the emerging opportunities in the commercial space sector, and iv) capitalizing on the emerging opportunities in human subjects research.

## Graphical abstract

**Figure:**
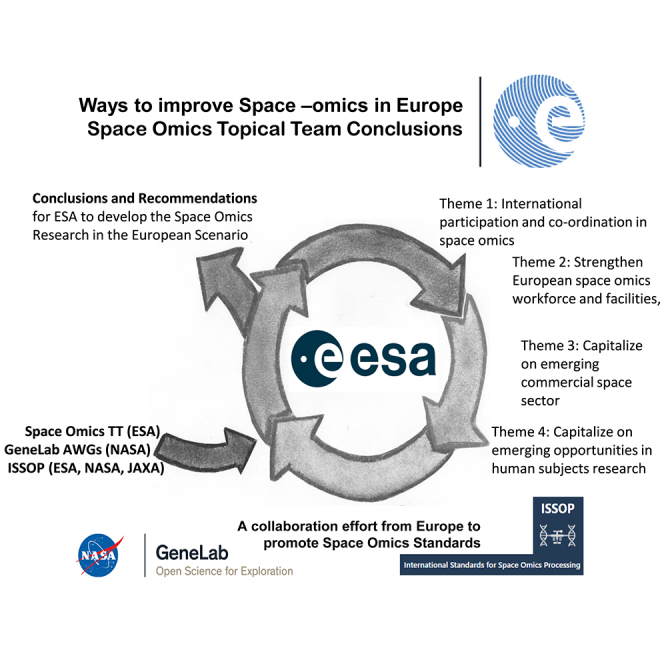

## Introduction

With the launch of Artemis missions to the moon, the consolidation of the company SpaceX and other private ventures as an alternative to National Aeronautics and Space Administration (NASA) launchers, and the replacement of the International Space Station (ISS) by several research-oriented commercial space stations toward the end of this decade, we are just at the gates of a new era of space research. In fact, the scientific component of Artemis and other missions has acquired unprecedented importance. Within life sciences, molecular biology, genomics, and personalized medicine methods have progressed exponentially, and such techniques have been applied mostly in lower Earth orbit in experiments onboard the ISS. Consequently, spaceflight and space biology research will become significantly more important during the next decade. To support space omics, NASA, European Space Agency (ESA), and Japan Aerospace Exploration Agency (JAXA) communities have promoted several initiatives, such as GeneLab led by NASA,^1^ the Space Omics Topical Team funded by ESA,^2^^,^^3^ and International Standards for Space Omics Processing (ISSOP^4^) that integrates a multi-national force of scientists interested in sample processing standardization and metadata normalization of space omics research, including those from JAXA.

The European life science community in space research has always been limited in numbers but fully devoted to microgravity research, leading international progress in particular model organisms. In the last fifteen years, the financial crisis, together with the emergence of private partners for NASA, made ESA-related researchers vulnerable to the lack of crewed mission launch capabilities. This has particularly affected the space omics research in which ESA-funded activities were reduced. As a result, European Principal Investigators depended on collaborations with international colleagues to keep the pace in their research goals, in some cases with outstanding results, but undermining European independence.

Space research careers at the university level should be encouraged in Europe. Several Master’s courses have been hosted by ESA, the European Low Gravity Research Association (ELGRA), and the International Space University (ISU) in recent years. However, the low number of students involved and the lack of a clear path for those students to pursue a career in the field reduces the impact of these academic plans. To retain talented young scientists and pursue excellence in space research, it is necessary to establish a strong network of European space biology research laboratories to promote student exchanges, from bachelor to post-doctoral level, as foundations to build a scientific environment that retains and attracts new researchers, particularly young post-doc researchers that will constitute the future of space research in Europe. Moreover, the timeline for progression of the career path in space research constitutes another major problem faced by junior researchers. To get permanent positions in laboratories in Europe requires an updated outstanding track record. However, spaceflight experiments require several years (sometimes decades) to be complete, from study conceptualization to the analysis of the results and publishing large amounts of data/studies, which then impacts the chances to attract funding, be part of evaluation panels, supervising students, and therefore, the ability to achieve a stable research position. Also, the lack of reproducibility and relative value of ground-based simulation facilities complicates obtaining clear and unquestionable results by space researchers. Choosing the space research path is choosing a hard path. Therefore, most junior researchers usually change their research topics to research fields, in which high-quality articles can be obtained at a faster pace. We should encourage junior life scientists in other fields to at least keep a “scientific arm” in spaceflight research.

Now, we encourage the European research community to capitalize on existing capacities to boost space omics research via maintaining our collaboration/s with NASA GeneLab via ISSOP and similar international endeavors, but to also build our own facilities and capabilities in ESA member states in order to compete with NASA and other space agencies for attracting the new generation of space scientists. At the same time, it is important to work in international research announcements to maximize the results of large, reference experiments required to be performed in key model systems. ESA has provided a strategic roadmap for the future, but the continuous redefinition of those working plans, with little call for opportunities for scientists, does not allow concrete actions. On the contrary, NASA is promoting Technology and Integrated Discipline Engineering Services, including a continuum of support to the space research community including spaceflight opportunities, funding for laboratory activities, and even data processing support via GeneLab.

As an ESA-funded Space Omics Topical Team, we have previously highlighted how the different funding scheme/s within the European Union (EU) is complex.^3^ This impacts the productivity of each country in the context of space omics research. To alleviate this, national and ESA programs supporting flight operations and science exploration should provide a predictable and sustainable funding landscape. This would help to promote and encourage collaborations among eligible countries as well as with other international space agencies and research centers. In our review of European space omic activities to date, we highlight a delay in incorporating more emerging/novel omics technologies to space research, with a dominance of transcriptomics (95% microarray versus studies versus 5% RNA-seq) versus other omic approaches (e.g., proteomics, metabolomics).^3^ Table 1 includes a comprehensive list of the European contributions to Cell Press collections in 2020 and 2022, which provides an up-to-date status of space omics research in Europe.

**Table 1:** Full list of European-led articles

Journal	Article Type	Title		First Author	Corresponding Author	DOI
iScience	Backstory	Building the Space Omics Topical Team to boost European Space Researchers’ role in the international consortia redefining spaceflight-generated datasets		Raúl Herranz	Raúl Herranz	https://doi.org/10.1016/j.isci.2022.104868
iScience	Review	Space omics research in Europe: contributions, geographical distribution, and ESA member state funding schemes		Colleen S. Deane	Raúl Herranz	https://doi.org/10.1016/j.isci.2022.103920 (∗)
iScience	Review	Recent transcriptomic studies to elucidate the plant adaptive response to spaceflight and to simulated space environments		Aranzazu Manzano	Aranzazu Manzano	https://doi.org/10.1016/j.isci.2022.104687
iScience	Review	Multiscale modeling in the framework of biological systems and its potential for spaceflight biology studies		Andrew Millar-Wilson	Gary Hardiman	https://doi.org/10.1016/j.isci.2022.105421
iScience	Article	Loss of physical contact in space alters the dopamine system in *C*. *elegans*		Surabhi Sudevan	Nathaniel J. Szewczyk	https://doi.org/10.1016/j.isci.2022.103762
iScience	Article	Muscle atrophy phenotype gene expression during spaceflight is linked to a metabolic stress crosstalk between the liver and the muscle in mice		Geraldine Vitry	Willian A. da Silveira	https://doi.org/10.1016/j.isci.2022.105213
iScience	Article	Optimization of RNA extraction for bacterial whole-transcriptome studies of low-biomass samples		Tom Verbeelen	Felice Mastroleo	https://doi.org/10.1016/j.isci.2022.105311
Heliyon	Article	The Maleth Program: Malta’s first space mission discoveries on the microbiome of diabetic foot ulcers		Christine Gatt	Joseph Borg	https://doi.org/10.1016/j.heliyon.2022.e12075
Patterns	Perspective	Routine omics collection is a golden opportunity for European human research in space and analog environments		Henry Cope	Nathaniel J. Szewczyk	https://doi.org/10.1016/j.patter.2022.100550
Cell Reports Methods	Review	Challenges and considerations for single-cell and spatially resolved transcriptomics sample collection during spaceflight		Eliah Overbey	Stefania Giacomello	https://doi.org/10.1016/j.crmeth.2022.100325
iScience	Review	*Caenorhabditis elegans* in microgravity: An omics perspective	Amanda Scott		Colleen S. Deane	https://doi.org/10.1016/j.isci.2023.107189
iScience	Perspective	Enhancing European capabilities for application of multi-omics studies in biology and biomedicine space research	Aranzazu Manzano		Raúl Herranz	https://doi.org/10.1016/j.isci.2023.107289 (∗)
Cell Systems	Letter	Revamping space omics in Europe		Pedro Madrigal	Raúl Herranz	https://doi.org/10.1016/j.cels.2020.10.006 (∗)
Cell	Article	comprehensive multi-omics analysis reveals mitochondrial stress as a central biological hub for spaceflight impact		Willian A. da Silveira	Afshin Behesthti	https://doi.org/10.1016/j.cell.2020.11.002
iScience	Article	Comparative transcriptomics identifies neuronal and metabolic adaptations to hypergravity and microgravity in *Caenorhabditis elegans*		Craig R.G. Willis	Tim Etheridge	https://doi.org/10.1016/j.isci.2020.101734
iScience	Article	The importance of earth reference controls in spaceflight -omics research: Characterization of nucleolin mutants from the seedling growth experiments		Aranzazu Manzano	Raúl Herranz	https://doi.org/10.1016/j.isci.2020.101686
iScience	Article	Cell-free DNA (cfDNA) and exosome profiling from a year-long human spaceflight reveals circulating biomarkers		Daniela Bezdan	Chris Mason	https://doi.org/10.1016/j.isci.2020.101844
iScience	Article	NASA GeneLab RNA-seq consensus pipeline: Standardized processing of short-read RNA-seq data		Eliah Overbey	Jonathan Galazka	https://doi.org/10.1016/j.isci.2021.102361 (∗)
Patterns	Perspective	A new era for space life science: International Standards for Space Omics Processing		Lindsay Rutter	Raúl Herranz	https://doi.org/10.1016/j.patter.2020.100148 (∗)
Cell Press 2020 Bundle: The Biology of Spaceflight: https://www.cell.com/c/the-biology-of-spaceflight Cell Press 2022 Bundle: Space Omics in Europe: https://www.sciencedirect.com/journal/cell-reports/special-issue/10K54CFNC0X						

(as first or corresponding author/s) included in “The Biology of Spaceflight” 2020 and “Space Omics in Europe” 2022 Cell Press Bundles. Articles signed by the full GeneLab Analysis Working Groups (AWG), International Standards for Space Omics Processing (ISSOP), or ESA-funded Space Omics Topical Team (TT) consortia are indicated (∗).

The ISSOP consortium has highlighted that the reliance of space biologists leveraging omics approaches is increasing.^4^ One reason is that performing omic analysis on pre-collected and correctly stored samples is possible and maximizes the knowledge gained from spaceflight experiments. Therefore, with a combination of multiple omics approaches, traditional and novel sequencing, and sophisticated downstream bioinformatics tools, research could achieve more precise, robust, and significant conclusions on the impact of different parameters that condition biology and life in the space environment. Afterward, following protocols for analysis standardization and dissemination of these discoveries, these data provide actionable scientific insights that enhance our comprehension of the (patho)physiology and biology during short- and longer-term space travel.

## Recommendations

**Figure 1:**
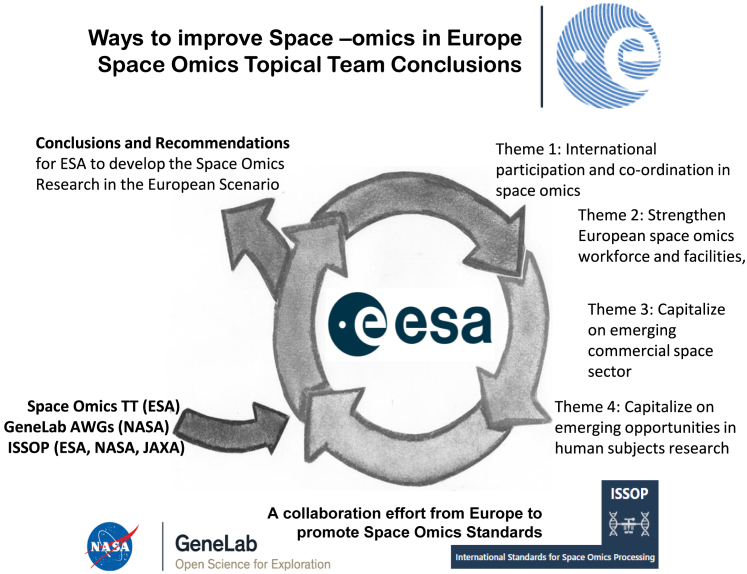
Working topics and methodology to achieve the recommendations to improve European space omics research

The following section represents recommendations to ESA regarding specific topics chosen by the ESA-funded Space Omic Topical Team and their works in the context of international efforts (NASA GeneLab Analysis working groups and ISSOP as described in Figure 1). To identify the elements that should be more urgently addressed to boost space omics research in Europe, we performed a Strengths, Weaknesses, Opportunities, and Threats analysis, with the aim to highlight existing advantages, raise awareness of limitations, and suggest actionable solutions.

### International cooperation and standardization

European researchers, due to their leadership coordinating international space research consortiums from major European research institutes, pioneered studies using omics data to assist in the comprehension of the cellular events induced by the extraterrestrial environment.^3^ Some cases of synergistic partnerships are ESA and CLAIRE, Europe’s largest artificial intelligence research network.^5^ Furthermore, European scientists in space have access to state-of-art facilities and resources such those available in the European Molecular Biology-European Bioinformatics Institute (EMBL-EBI) located in Hinxton (UK), which in addition to research activities maintains services and databases such as Ensembl, UniProt, AlphaFold, BioStudies, MGnify, Expression Atlas, or PRIDE, to name only a few. Currently, Europe provides a wide range of great opportunities for synergies and networking at a regional, national, and European level for states that are both members of ESA and EMBL. This is enhanced by the state-of-the-art sample and data collections, in addition to the outstanding bioinformatics expertise available, the continuous crosstalk and exchange of initiatives with GeneLab and ISSOP, as well as the prominent engagement in international space biology studies. As a result, European scientists provide significant contributions on data and analysis standardization,^6^ as well as engagement in international agreements for standardization from other fields. Hence, developing standard operating procedures across all member states of ESA would contribute to perform more sophisticated comparisons of datasets to be applicable in translational studies.

Currently, not many relevant grant opportunities or funds from ESA, other European programs of research or national calls for space omics in Europe exist. Some members of the European space biology community have suffered from cancellations in already approved spaceflight experiments due to budget cuts and the recent calls for ideas will also lead to a repository of experiments that will be prepared during years without any commitment to be launched by ESA. There also seems to be no coordination at international level from ESA or their state members. This leads to little international cooperation in connection with omics applications and ESA does not seem to develop research programs to change this current ecosystem in space biology. As an example, currently, there does not exist a counterpart laboratory to NASA GeneLab in Europe. Consequently, and to capitalize on the application of (multi-)omics approaches in space biology, we recommend and encourage ESA to significantly increase its funding investment and initiative that supports omics-based investigations and the generation of a European space omics database. This recommendation is not only regarding physical institutions but also “virtual” institutes focused on space-related projects, particularly if the critical mass of researchers is evenly distributed in Europe.

Our Topical Team also suggests that ESA should take into consideration that there exists a chance for ESA to take a leading role in space biology publications due to the excellent research centers in all member states. However, unlike USA-based scientists that have been fully supported by NASA and federal administration, Principal Investigators in Europe are mostly isolated without a supportive critical mass of other scientists in their institutions or in other nearby research centers, which prevents multi-disciplinary coordinated investigations. Problems stemming from no multi-national coordination are for example lacking standards and certification processes for commercial spaceflight, conflicting national interests and regulations, the problem of increasing space debris and lacking planetary protection, as well as the language barrier when developing, translating, and adoption of standard operating procedures (SOPs). The latter has in the past been more difficult than developing SOPs. This could lead to other countries that are already very active on the subject taking over decisions. A final point to consider is that there exist different timelines and objectives from the private and public sector.

## Strengthening European capacities: Human resources

In 2008, the United Nations Office for Outer Space Affairs (UNOOSA) developed a core education curriculum to its affiliated centers. This curriculum was developed containing five major fields of space applications, being: i) Satellite Meteorology and Global Climate, ii) Satellite Communications, iii) Space and Atmospheric Science, iv) Remote Sensing and Geographic Information Systems, and v) Global Navigation Satellite Systems.^7^ Although an important step to increase standardization and quality of space science education around the world in specific courses, the incorporation as part of the formal education curricula was not achieved for Space Biology. We recommend extending the teaching of Space Biology and Space Medicine onto the highest education levels and highlight the important addition of space omics in the curricula.

In Europe, we have a multi-faceted and strong life science community. This is reflected in various international collaborations inside and outside of the EU over decades with significant impact on, for example, the rapid development of vaccines against COVID-19. The EBI (part of the EMBL) is the principal source for training on big data in biology and together with the European Molecular Biology Organization (EMBO) provides training on omics experimentation and bioinformatic analysis in the continent.^8^^,^^9^ There are also numerous educational programs focusing on space life sciences, e.g., summer courses funded by ESA for more than two decades ago that evolved into the summer university currently promoted from the ESA Education office in Belgium. These summer courses engage students into Science, Technology, Engineering, and Math careers but there is not a next step in their curricula should they wish to continue engaging space biology. However, the only formal training on space omics that the authors are aware of occurs as part of ISU Master of Space Studies, being delivered continuously since the academic year of 2020/2021. In contrast, in the USA, NASA GeneLab has been organizing GeneLab for High Schools since 2017 and GeneLab for Colleges and Universities pilot program started in May 2022.^10^^,^^11^ To develop space omics in Europe, we recommend ESA to implement similar initiatives at the continental level and in a joint effort with EMBO/EMBL and other interested academic partners (e.g., Universities). We also recommend the theme of space omics to be included in future ELGRA meetings and educational activities.

Regarding a career in space research, or more specifically space omics, multiple points were raised aiming to enrich Europe’s curriculum and availability. Since space omics is a relatively young branch of science in the EU (compared to other areas^3^), there are missing or under developed areas that would facilitate the implementation of multi-omics approaches in the study of space biology. As an example, there are very limited research career options for post-graduates due to the lack of work positions in academic institutions and industry. This is exacerbated by the current lack of a readily available website/s listing European Space Education possibilities. Another factor is that there are still not enough experts in space bioscience who can teach and support respective careers. Even if people educate themselves in these topics, a “Brain-drain” of European scientists is being observed because of the mentioned precarious working conditions (e.g., fixed-term contracts) or unemployment for those not being able to leave their home country. Missing experts could lead to non-certified space biology education courses, which could further lead to dilution of educational standards.

Some of these problems could be prevented if ESA and the EU countries make funding available to enhance the academic and industry ecosystem of the space sector. In the academic fields, this is especially necessary to increase the critical mass of pre-doctoral and post-doctoral positions, while offering realistic prospects for the future. This can only work if worldwide recognized scientists in space biology hold academic positions in universities and schools. For increasing the offer for groundwork research applying omics, public and/or private academic institutions in potential coordination with the space industry would be required to organize BSc/MSc degrees in space research merging life science with computer science and bioinformatics. In addition, the Topical Team recommends the initiation of interdisciplinary research projects through members of the Topical Team, the support of the UNOOSA initiative “Access to Space for All”,^12^ and the promotion of the “Spin/Fly Your Thesis” program.^13^

On the recent “Terrae Novae 2030+ Strategy Roadmap”, ESA stated its aim to maximize opportunities for the life science community^14^ having defined previously “Understanding the Impact of Gravity on Biological Processes, Cells and Organisms” as one of the agency roadmaps.^15^ Therefore, as a Topical Team, we conclude that providing young researchers with a wider range of options like space biosciences is crucial to make Europe an attractive and modern place for space-related science and to allow ESA to fulfill its declared goals.

Stable careers can be made possible through connections between public and private sectors. The “Business In Space Growth Network Life Science Industry Accelerator” initiative seems to be a good start, but its impact is yet to be determined.^16^ However, facilitating outreach to a large number of member states with different languages can be very challenging. It could be leveraged that there is widespread availability of English speakers in Europe’s educational systems. Problems hindering that approach are missing education programs thus leading to a one-only approach, competition from multiple sources (Organizations, Universities, Countries), and an increasing elitism in the scientific community.

### Strengthening European capacities: Infrastructures for space omics

Europe has a solid network of infrastructures with centers in the Netherlands, Italy, Germany, England, and Spain, connecting a wide scientific community of researchers with expertise in very diverse fields and with collaborations and active international projects.^3^ This community is made up of research groups with extensive experience in space omics that integrate cellular and molecular biologists, physiologists, microbiologists, statisticians, and bioinformatic experts,^2^ which has led to the creation of consortiums made up of topical teams, as a first step for research in space omics to be integrated into existing facilities, such as European Space Research and Technology Center (Netherlands) or Science Data Centers (Madrid). It is unclear whether ESA will promote the future construction of a single building for cutting-edge space biology research in Europe, with the additional mission to become a reference in implementing standardization protocols worldwide as well as coordinate key collaborative initiatives with public and commercial space industry for Europe to capitalize the race to space. Related to this, the lack of development and integration in the current databases from space biology experiments at ESA constitutes an opportunity to generate a large database that includes omics, the details of the sample repository, images captured during orbital activities, as well as valuable metadata information captured from in flight operations at User Support and Operations Centers facilities.

For this endeavor, the experience of ELIXIR (https://elixir-europe.org) on developing life science resources across Europe could be crucial. The goal of ELIXIR is to coordinate these resources so that they form a single infrastructure and bring together more than 220 European organizations. In summary, there is excellent expertise in Europe from which ESA could benefit from to manage the huge increase of space life sciences data that is expected in the future, and to establish the necessary bioinformatics infrastructure, taking advantage of existing infrastructure. ESA Science Data Centers should collect inputs from ESA and European principle investigators to achieve a similar level of integration to other databases including GeneLab (space omics repository, not existing at ESA), ALSDA (Ames Life Science Data Archive, equivalent to ESA ERASMUS archives in Europe), BSP (Biospecimen Sharing Program), NBISC (NASA Biological Institutional Scientific Collection), and PSI (Physical Sciences Informatics). This does not necessarily mean to establish new databases with added value, but to create stable links and cooperation between the existing archive at ESA and both European (EMBL/EBI) and American (GeneLab) sources of space omics datasets. We also propose to assume a major role in the development of European sample biobank (for example, the UK Biobank) to integrate European samples/data into NASA repositories in case an ESA/NASA agreement is not feasible.

The analysis of the whole genome data is not trivial and requires computing infrastructure and databases, as well as advanced bioinformatics solutions and expertise for large-scale data analysis. Prioritization in large-scale space technology research programs is inevitable and is already happening in NASA-supported research. NASA established GeneLab, an interactive and publicly available resource where scientists can store, share, and analyze the data from space-related experiments (Ray et al. 2019). JAXA also opened the Integrated Biobank for Space Life Science (ibSLS, https://ibsls.megabank.tohoku.ac.jp/), an interactive omics database and sample sharing system of JAXA’s space mouse missions.^17^ ESA is currently defining a new scientific program and it is a unique moment to create new tools and lead new initiatives in space sciences for the whole genome data. Storing the data on European servers is important for several reasons. Firstly, it helps ensure that the data are subject to EU regulations and laws, such as the General Data Protection Regulation, which are designed to protect the privacy and security. Secondly, it reduces the risk of the data being accessed or hacked by unauthorized third parties, including those who may mishandle the data. Thirdly, it helps support the EU’s and ESA’s efforts to promote growth and competitiveness of the European cosmic sector by providing a secure and reliable infrastructure for businesses to operate in.

While the existing resources contain mainly bulk genomic profiles, it would be complementary to create an additional infrastructure dedicated to single-cell and spatial analysis.^18^ Although bulk analyses have broad utilities, single-cell analyses provide a more precise understanding of the cell-type-specific adaptations to environmental challenges as demonstrated for instance by the Human Cell Atlas. There is a need for a wide initiative that is going to advance space science and provide means to recognize and trace outer space-related abnormalities at the single-cell and spatial level.

Understanding the body’s response to various external factors, such as those occurring in microgravity, requires a thorough understanding of the reactions taking place in individual cells. This leads to the collection of huge amounts of data and specialized computational analysis using machine learning and artificial intelligence methods. European scientific groups have been successfully conducting both molecular and computational research at the single-cell level on Earth^19^ and spatially resolved transcriptome profiling.^20^^,^^21^

Now, ESA has the chance to capitalize and could lead the adaptation of existing methods and the development of space-specific single-cell methodologies and pipelines that could be integrated into a robust, standardized approach. In addition, to provide interpretable and accountable computational solutions and allow researchers to benefit from the already existing datasets, machine learning and artificial intelligence methods should be considered. Such a new dedicated platform could provide a technical framework for integrating various types of molecular and imaging datasets with health records facilitating joint analyses. This should also introduce standards regarding storing and pre-processing space-related data. The development of such platform at the single-cell level combined with machine learning and artificial intelligence solutions would build solid foundations for European space genomics.

### Maximizing opportunities afforded by commercial space

Access to space constitutes an essential opportunity to carry out key experiments to advance our understanding of the physiological adaptations to altered gravity environments. Thus, Europe historically has access to payloads on foreign missions, including Roskosmos (currently banned due to international conflicts between Russian and Ukraine). There are no efforts to ban experiments with other specific partners. In addition, ESA offers potentially diverse opportunities (sounding rockets, ice cube, ISS, etc.) and there are a rising number of funding opportunities available with international organizations like ISS National Laboratory or the wetlab2 community. All this provides the opportunity for researchers to integrate their experiments into funded flights, which in turn encourages more and more scientists to participate in space research. Connecting financial support to technological advancements and industrial growth also encourages the rise of private rocket manufacturing entities. One such example is “The Exploration Company,” a German European spacecraft manufacturer, which produces the Nyx space capsule. This approach makes flight missions, primarily those centered around the ESA, more attainable. All of this leads to many new commercial spaceflight industry partners that could provide Europe exclusive rights to a fair share of payloads, which every European researcher could access regardless of costs and waiting times.

Despite all this, European researchers find it extremely difficult to carry out space experiments because flight opportunities and project funding are not linked and calls are not well publicized, causing a significant dependency on collaborations to obtain funds that support experiments during space missions. Moreover, European scientists face other limiting factors such as the long timeline, sometimes years, of a space experiment as well as the geographical location of Europe, which is not the most suitable for launching rockets and limits the chances to launch space projects. All this suggests that Europe needs a significant increase on their own budget for research on the ground, the space, and flights that support their independence from other major world powers, lead partnerships with other international institutions, and protect the intellectual property rights that will secure their return on investments as royalties.

Although ICT and Health that support life sciences are both high priority for the EU, space experimentation requires significant funding and scientific experts in very diverse fields integrated in these themes. Another observation is that digitalization of the Life Sciences & Health Sector is a priority for the EU. The low number of ESA-funded research projects, together with the impossibility of expanding laboratory infrastructures for space biosciences research in canonical, non-space specific research centers, prevents attracting space bioscience experts to Europe. It is also worth mentioning the long waiting period for access to ESA infrastructures, particularly to spaceflight opportunities. Therefore, we urge to promote space research to the same level as China and the USA to prevent Europe from being left behind in the standardization of space omics research.

With the beginning of 2022, NASA have been prioritized funding opportunities for public-private partnerships with the goal of expanding capabilities and opportunities in space. ESA has responded with a similar effort by funding ESA Commercialization Gateway (https://commercialisation.esa.int/) with the ambition to make Europe a space commercialization hub to launch and grow global space companies in technology and biomedicine sector. ESA Commercialization Gateway does not only provide its own space incubator and investor network but also invites researchers to apply for Commercial Applications enabled by Space Environments grants in collaboration with commercial spaceflight providers such as yuri GmbH, the space exploration company, and others.^22^

In the last few years, more and more grants have emerged to support this journey; ESA’s Technology Transfer and Patent Office turned to the Discovery element of ESA’s Basic Activities. Through the Open Science Innovation Platform, the Discovery element issued a call for ideas on innovative ways to commercialize a collection of promising ESA patents.

### Maximizing opportunities afforded by human space omics

Collecting omics routinely from humans entering space presents a golden opportunity for European space research; policy pertaining to human omics studies in spaceflight will be an important issue to reach a consensus on among European and international stakeholders. The NASA Twins study showcased the feasibility of collecting omics data from astronauts throughout the whole course of a long-duration mission, including in-flight measures with minimally invasive biopsies such as blood, and non-invasive harvesting of samples like urine.^23^ Similar human multi-omic studies are now occurring internationally through commercial ventures (e.g., SpaceX Inspiration4 and Polaris Dawn) and via space agencies (e.g., JAXA Cell-Free Epigenome). It is essential for Europe to rise to this emergent opportunity, leveraging considerable experience and expertise in designing human performance studies and model organism omic studies in space.^3^ Every European entering space, either via government or commercial means, should now be viewed as a potential test subject from which omics can be collected with their informed consent and appropriate ethical procedures in place. Additionally, these collection protocols can be aligned with those from analog studies, to allow for high fidelity comparison between experiments.

The opportunity of human space omics is 2-fold. Firstly, such studies could improve medical risk quantification and occupational healthcare for astronauts, even in the immediate term. For example, if deployed today, pre-flight genomic screening of astronauts could be used for pharmacogenomics, optimizing the drugs prescribed to individual crew members to improve performance and avoid adverse drug reactions. The potential for pharmacogenomics is evident from a European study which found approximately one-third of the drugs on the ISS to be affected by genetic differences in metabolizing enzymes.^24^ Secondly, human omics studies in space could accelerate the understanding of the impact of spaceflight directly on human biology, such as by elucidating mechanisms of spaceflight-associated neuro-ocular syndrome. Thus, collecting human space omics is immediately actionable while also presenting a path for future advanced space biology research. Thorough discussion regarding appropriate policy today could lead to the generation of datasets designed with future research and analysis capabilities in mind. For example, by collecting correlated metadata and functional data (e.g., exercise performance) alongside omics, machine learning and artificial intelligence approaches could be applied to identify significant relationships between spaceflight-induced omic changes and phenotypic changes.

In order to deploy a routine program, we must consider practicalities and logistics in detail, to design a cost-effective and standardized program. As such, in a recent manuscript from members of the ESA-funded Space Omics Topical Team, a number of considerations were introduced and a set of questions were suggested to motivate further discussion among experts and stakeholders.^25^ The first subset of questions aims to initiate discussion of the possible collection routines from astronauts and spaceflight participants, decide on the omics data to collect, from which samples, and at which time points. These questions and some initial thoughts are listed below.

(1) **Which omic types and technologies have the highest potential for scientific return and clinical actionability (with additional consideration to multi-omic combinations)?** - Genomic sequencing is likely to be the most immediately clinically actionable. Other omic types, including transcriptomics, microbiomics, and proteomics have potential clinical applications and may be useful for monitoring the effect of spaceflight throughout the mission.
( 2) **Which sample types are the most practical to collect (i.e., cost, sample processing procedures, and invasiveness)?** - For frequent sampling, particularly in-flight, non-invasive measures including urine, saliva, skin swabs, and stool will be useful.^25^ Blood samples are also a great option for omics, due to being minimally invasive and well established for spaceflight collection and medicine on Earth.
(3) **What metadata would be the most useful and practical to standardize alongside omics collection (e.g., physiological, environmental, and lifestyle)?** - Spacecraft environmental data will be essential as will regular performance data, such as those collected via NASA’s Spaceflight Standard Measures Program (e.g., cognition, exercise, diet).
(4) **At which time points should omics be collected?** - Pre-flight screening is recommended, and multiple pre-flight time points for dynamic omics are important for building a normalized baseline. Multiple in-flight omic time points are useful for monitoring change throughout the flight, including within the first few days where significant physiological changes already occur.^26^ Post-flight data will be important for monitoring recovery and lingering effects.
(5) **How can the artificial intelligence-readiness of the generated datasets beoptimzed?** - Data and metadata should be standardized between human space omic studies and stored in machine-readable formats. Due to personal data challenges, it seems logical to ensure support for federated learning.

The second subset of questions pertains to the ethical, legal, and regulatory considerations of designing policy for European spaceflight omics studies in humans. Suggested questions for discussion and some initial recommendations are shown below.

(1) **What is the potential identifiably of different omic types, in the specific context of astronauts?** - Astronauts are particularly easy to identify since they are a small cohort of public figures, with phenotypic information on individuals readily available online. This easily accessible phenotypic information could be combined with omic information through linkage attacks, to identify individuals and breach their privacy.^25^ Genomic data are likely to hold higher identification risk compared to other omic types, yet other data types and combinations cannot be considered to be low risk without thorough analysis.^27^
(2) **How should ESA go about obtaining meaningful informed consent for omics research?** - Broad consent is often used for omics research (e.g., UK Biobank), since analysis of omics data is not always hypothesis driven and future data uses are sometimes unknown. However, efforts should be taken to educate subjects as much as possible on the potential risks of having their omics data collected, and standard procedures such as oversight via ethical review boards should be followed.
(3) **How should ESA handle potential ethical issues, such as incidental findings and discrimination?** - Similar to omic initiatives on Earth, clear policy which includes genetic counseling will be required for handling incidental findings, and regarding discrimination, focus should be given to using findings from omics to tailor countermeasures for individual crew members during spaceflight, especially if they are at a higher risk for a medical incident. An ESA Topical Team has recently discussed the opportunities for personalized medicine in space.^28^
(4) **How should data storage and data sharing between ESA’s European and international network be handled?** - Data sharing is desirable to enable open-science efforts, and NASA GeneLab is well established for data sharing of non-sensitive space omics data. However, most of the omics data are likely to be highly sensitive, so uploading sensitive data to a controlled access biobank, such as the UK Biobank and Federated European Genome-phenome Archive, to provide access only to approved researchers may make sense. Federated approaches, with the human omics data stored in local secure European biobanks, may also serve as an appropriate option for European-wide and even international data sharing.^25^

### Conclusions

Several potential weaknesses and threads for European researcher’s participation in the space omics discipline development at a global level have been identified for which we provide recommendations to minimize these risks (Table 2). Even if space omics is now specifically included among the topics of interest in biology research goals of ESA roadmaps,^29^ a specific effort should be made in this area to align European researchers’ capabilities with those on other space agencies around the globe.

**Table 2:** Top 10 issues detected by our Strengths, Weaknesses, Opportunities, and Threats (SWOT) analysis and our recommendations to solve or minimize their impact in European space omics research

Potential risk	Recommendations
No co-ordination at international level to align ESA and state member policies.	A common policy on data sharing, including human subject’s data, should be established at ESA level.
Members from European research institutes remain isolated in non-thematic centers.	A virtual Space Omics research center (network) should be established.
A specific space omic/database community (similar to GeneLab) is missing in Europe.	Adoption of a common ESA database and funding computational analysis of deposited space omics data should be considered.
Lack of omics standards for principal investigators in the ESA calls for opportunities	ESA should consider the adoption of omics standards, perhaps utilizing the International Standards for Space Omics Processing or similar international consortia recommendations. In concert with commercial spaceflight industry to implement them
ESA has lower launch capabilities for spaceflight experiments than other agencies and the associated costs remain high.	ESA should embrace the emerging commercial spaceflight sector as a route to obtain more space omics data.
Single state members have different mechanisms to fund space scientists.	ESA should consider mechanisms to ensure funding for multi-national research collaborations/teams.
Limited career options and opportunities for postgraduates.	ESA should consider mentorship schemes and support for individual researchers, in addition to the traditional project-based funding schemes, for example in collaboration with the commercial spaceflight industry
Brain-drain of European researchers.	Establishment of a space omics research program at the international level via partnerships with NASA and JAXA at a minimum, and consideration of space biology as a fundable space activity in EU calls.
Long process from project concept to execution and publication of results.	Better use of ground-based facilities program to prevent research arrest period between spaceflight opportunities. For example, omics collection in human subjects in analog environments.
The strong life science community in Europe is not interested in Space Biology.	ESA sponsoring space omics researchers at life science-focused conferences in Europe may help recruit interested researchers.

## Consortia

The ESA-funded Space Omics Topical Team aims to support the use of omics research among the ESA space biology community. The members of the Space Omics Topical Team are European affiliated scientists landing at the Space Biology research field as Principal Investigators from spaceflight biological experiments or as bioinformatics experts. The list of members contributing to this article are Daniela Bezdan, Joseph Borg, Thomas Cahill, Eugénie Carnero-Diaz, Henry Cope, Colleen S. Deane, Timothy Etheridge, Stefania Giacomello, Gary Hardiman, Raúl Herranz, Natalie Leys, Pedro Madrigal, Aránzazu Manzano, Felice Mastroleo, F. Javier Medina, Jakub Mieczkowski, Manuel A. Fernandez-Rojo, Keith Siew, Willian A. da Silveira, Nathaniel J. Szewczyk, Stephen B. Walsh, and Silvio Weging.
